# Minimization of ragweed allergy immunotherapy costs through use of the sublingual immunotherapy tablet in Canadian children with allergic rhinoconjunctivitis

**DOI:** 10.1186/s13223-023-00758-7

**Published:** 2023-01-18

**Authors:** Anne K. Ellis, Douglas P. Mack, Rémi Gagnon, Eva Hammerby, Sheena Gosain

**Affiliations:** 1 Division of Allergy & Immunology, Department of Medicine, Queen’s University, Kingston, ON Canada; 2 Department of Medicine, McMaster University, Hamilton, ON Canada; 3 Halton Pediatric Allergy, Burlington, ON Canada; 4 Clinique Spécialisée en Allergie de La Capitale, Québec, QC Canada; 5 ALK-Abelló A/S, Hørsholm, Denmark; 6 PDCI Market Access, Inc, Ottawa, ON Canada

**Keywords:** Cost, Ragweed, Subcutaneous immunotherapy, Sublingual immunotherapy, Tablet, Allergic rhinoconjunctivitis, Children

## Abstract

**Background:**

Allergy immunotherapy (AIT), in the form of subcutaneous immunotherapy (SCIT) with alum-precipitated aqueous extracts, SCIT with a modified ragweed pollen allergen tyrosine adsorbate (MRPATA; Pollinex^®^-R), or a sublingual immunotherapy (SLIT)-tablet are options for the treatment of ragweed pollen allergic rhinoconjunctivitis (ARC) in Canadian children. A cost minimization analysis evaluated the economic implications of the use of the ragweed SLIT-tablet vs SCIT in Canadian children with ragweed ARC.

**Methods:**

A cost minimization analysis was conducted comparing the short ragweed SLIT-tablet, 12 Amb a 1-U, preseasonally with preseasonal ragweed SCIT, annual ragweed SCIT, or MRPATA. The analysis was conducted over a time horizon of 3 years from a public payer perspective in Ontario and Quebec. Resources and costs associated with medication and services of healthcare professionals were considered for each treatment. The resource and cost input values for the model were obtained from published literature and validated by Canadian clinical experts in active allergy practice. A discount rate of 1.5% was applied. Several scenario analyses were conducted to determine the impact of many of the key base case assumptions on the outcomes.

**Results:**

Over the total 3-year time horizon, the ragweed SLIT-tablet had a potential cost savings of $900.14 in Ontario and $1023.14 in Quebec when compared with preseasonal ragweed SCIT, of $6613.22 in Ontario and $8750.64 in Quebec when compared with annual ragweed SCIT, and $79.62 in Ontario and $429.49 in Quebec when compared with MRPATA. The ragweed SLIT-tablet had higher drug costs compared with the other AIT options, but lower costs for healthcare professional services. The lower costs for healthcare professional services with the ragweed SLIT-tablet were driven by the need for fewer office visits than SCIT. Scenario analysis indicated that costs were most impacted by including societal costs (e.g., costs associated with patient/caregiver travel and time lost). The potential cost savings of the ragweed SLIT-tablet versus SCIT and MRPATA was maintained in most scenarios.

**Conclusions:**

In this cost minimization analysis, the ragweed SLIT-tablet provided estimated cost savings from a public payer perspective for the treatment of ragweed ARC in Canadian children compared with SCIT or MRPATA.

**Supplementary Information:**

The online version contains supplementary material available at 10.1186/s13223-023-00758-7.

## Introduction

Allergic rhinoconjunctivitis (ARC) affects adults and children worldwide. In Canada, a nationwide survey of adults found that 20% of the surveyed population had been diagnosed with ARC [1]. The symptoms of ARC interfere with daily activities and sleep and are associated with poor concentration, fatigue, and reduced productivity [1, 2]. Children with ARC also have reduced school productivity and performance [2, 3]. This impairment in academic performance has been suggested to significantly affect long-term employment success and economic earnings [4].

Ragweed is one of the most common allergens associated with ARC in Canada and is most prevalent in Ontario and Quebec [5]. In a study across Canada, 28% of adults in Hamilton, Ontario, and 33% in Montreal, Quebec, were sensitized to ragweed [6]. Symptom-relieving medications such as antihistamines and intranasal corticosteroids can be used to treat the symptoms of ARC but have no impact on the disease itself. Alternatively, allergy immunotherapy (AIT) treatment modifies the pathogenic mechanisms that drive ARC, with a subsequent reduction in ARC symptoms and symptom-relieving medication use that can last years after stopping treatment [7–9]. In addition, AIT has been demonstrated to be a cost-effective treatment for ARC compared with symptom-relieving medication [10].

Allergy immunotherapy options for ragweed ARC in Canada include preseasonal subcutaneous immunotherapy (SCIT), using either alum-precipitated aqueous extracts or a modified ragweed pollen allergen tyrosine adsorbate (MRPATA; Pollinex^®^-R, Allergy Therapeutics [UK] Limited, Worthing, UK), and the short ragweed sublingual immunotherapy (SLIT)-tablet, 12 Amb a 1-U (Ragwitek^®^, ALK-Abelló A/S, Hørsholm, Denmark) preseasonally. MRPATA is a commercial SCIT preparation approved in Canada for the treatment of ragweed ARC in adults and children (ages 8 years and up) [11]. The ragweed SLIT-tablet was approved in Canada for adults in 2014 and for children (ages 5 years and up) in 2021 to reduce the signs and symptoms of moderate to severe seasonal short ragweed pollen ARC [12]. SCIT is an effective option for the treatment of ARC [13], but the need for multiple injections and frequent clinic visits increases the economic burden on healthcare resources and is inconvenient for patients and their caregivers. In contrast, if tolerated, SLIT-tablets can be taken at home after the first dose is administered under medical supervision in the clinic. SLIT is also considered to be safer than SCIT [14, 15]. Studies and patient surveys indicate that patients have a strong preference for SLIT over SCIT because of the convenience of at home administration and the more favorable safety profile [16–19]. For the same reasons, these preferences also translate to caregivers of children receiving AIT [19]. A cost minimization analysis was conducted to evaluate the economic implications of the use of the ragweed SLIT-tablet vs SCIT in Canadian children with ragweed ARC.

## Methods

### Cost minimization analysis

A cost minimization analysis was conducted to determine the cost impact of the ragweed SLIT-tablet, 12 Amb a 1-U versus other AIT comparators (e.g., ragweed SCIT prepared from alum-precipitated aqueous extracts or MRPATA) in Ontario and Quebec. A cost minimization analysis was chosen as the type of economic analysis because there are no available head-to-head efficacy and safety data between the ragweed SLIT-tablet and the comparators. It was conservatively assumed that the ragweed SLIT-tablet and the AIT comparators were therapeutically equivalent, despite evidence that SLIT has a more favorable safety profile than SCIT [14, 15]. The use of symptom-relieving medications for ARC symptoms was not included in the analysis based on the assumption that their use would be the same among the evaluated AIT options.

The analysis was conducted over a time horizon of 3 years, which is the minimum recommended duration of AIT treatment for seasonal pollens [8, 20]. The benefits and costs after completing 3 years of treatment were assumed to be the same for the ragweed SLIT-tablet and the AIT comparators. A public payer perspective relevant to Ontario or Quebec was adopted to estimate costs; therefore, no patient resources or costs (i.e., travel time/costs) were included in the base case model. A discount rate of 1.5% was applied in the base case model in accordance with Canadian economic evaluation guidelines [21].

The input values for the model were obtained from published literature and validated by Canadian clinical experts in active allergy practice.

### Model resource inputs

Medication resources and services of healthcare professionals were considered for each treatment included in the analysis. These resources for the ragweed SLIT-tablet, ragweed SCIT, and MRPATA over a 3-year treatment period are summarized in Table 1.

**Table 1:** Model resource inputs for medication and services of healthcare professionals in Ontario and Quebec

Resource	Ontario						Quebec					
	Ragweed SLIT-tablet	Preseasonal ragweed SCIT	Annual SCIT			MRPATA	Ragweed SLIT-tablet	Preseasonal ragweed SCIT	Annual SCIT			MRPATA
	Years 1, 2, and 3 each	Years 1, 2, and 3 each	Year 1	Year 2	Year 3	Years 1, 2, and 3 each	Years 1, 2, and 3 each	Years 1, 2, and 3 each	Year 1	Year 2	Year 3	Years 1, 2, and 3 each
Number of tablets	180	0	0	0	0	0	180	0	0	0	0	0
Number of treatment sets or maintenance vials	0	1	2	2	2	0	0	1	2	2	2	0
Number of 10 mL vials	0	0	0	0	0	1	0	0	0	0	0	1
Number of claims	6	1	2	2	2	1	6	1	2	2	2	1
Number of start-up visits	1	0	0	0	0	0	1	0	0	0	0	0
GP (5%)	0.05	0	0	0	0	0	0.05	0	0	0	0	0
Specialist (95%)	0.95	0	0	0	0	0	0.95	0	0	0	0	0
Number of follow-up visit	1	0	0	0	0	0	1	0	0	0	0	0
GP (10%)	0.1	0	0	0	0	0	0.1	0	0	0	0	0
Specialist (90%)	0.9	0	0	0	0	0	0.9	0	0	0	0	0
Number of titration visits	0	11	25	0	0	4	0	11	25	0	0	4
GP (10% SCIT, 80% MRPATA)	0	1.1	2.5	0	0	3.2	0	1.1	2.5	0	0	3.2
Specialist (90% SCIT, 20% MRPATA)	0	9.9	22.5	0	0	0.8	0	9.9	22.5	0	0	0.8
Number of maintenance visits	0	0	6.75	13	13	0	0	0	6.75	13	13	0
GP (95%)	0	0	6.41	12.35	12.35	0	0	0	6.41	12.35	12.35	0
Specialist (5%)	0	0	0.34	0.65	0.65	0	0	0	0.34	0.65	0.65	0
Number of consultation visits, with injection	0	11	31.75	13	13	4	0	11	31.75	13	13	4
GP (10% SCIT, 80% MRPATA)	0	1.1	8.91	12.35	12.35	0.8	0	1.1	8.91	12.35	12.35	0.8
Specialist (90% SCIT, 20% MRPATA)	0	9.9	22.84	0.65	0.65	3.2	0	9.9	22.84	0.65	0.65	3.2
Number of consultation visits, no injection	0	1	1	1	1	1	0	1	1	1	1	1
GP (20%)	0	0.2	0.2	0.2	0.2	0.2	0	0.2	0.2	0.2	0.2	0.2
Specialist (80%)	0	0.8	0.8	0.8	0.8	0.8	0	0.8	0.8	0.8	0.8	0.8
Nurse: time worked for injections, h	0	5.50	15.88	6.50	6.50	2.00	0	0^a^	0^a^	0^a^	0^a^	0^a^

*GP* general practitioner, *MRPATA* modified ragweed pollen allergen tyrosine adsorbate, *SCIT* subcutaneous immunotherapy, *SLIT* sublingual immunotherapy

^a^No nurse observation time was included because it is not covered by the Régie de l’assurance maladie du Québec

Medication resource use was assumed to be the same for Ontario and Quebec. Using information from the Health Canada product monograph [12] for the base case analysis it was assumed that the ragweed SLIT-tablet would be taken once daily during the pre-season (starting at least 12 weeks before the pollen season) and continued through the pollen season, for a duration of 6 months [22] This regimen was assumed to be repeated for years 2 and 3. There were 2 SCIT regimens compared in the analysis. The first SCIT regimen was pre-ragweed pollen season (preseasonal) monotherapy. The duration of preseasonal SCIT varies in practice from 8 to 13 weeks; a conservative estimate of 11 weekly preseasonal injections given during the titration phase, with 1 week between injections, and no yearly maintenance injections was assumed for the base case analysis. These assumptions were validated by Canadian clinical experts as representative of preseasonal SCIT treatment. The preseasonal SCIT regimen was assumed to be repeated for years 2 and 3. The second SCIT regimen was annual treatment, in which a base case of 25 weekly titration injections were assumed with 1 week between injections, followed by maintenance injections every 4 weeks. It was assumed that one 10 mL vial would last for 10 injections [23]. MRPATA was assumed in the base case to be administered by a healthcare provider in 4 weekly preseasonal injections. This regimen was assumed to be repeated for years 2 and 3.

The services of physicians and nurses are required in the assessment, prescribing, and administration of the ragweed SLIT-tablet, ragweed SCIT, and MRPATA. A key difference in healthcare resource use among the AIT options is that after the first dose is administered in the clinic, the ragweed SLIT-tablet is administered at-home. Therefore, for Ontario it was assumed that there would be one initial visit each year with a physician (95% specialist, 5% general practitioner) for the ragweed SLIT-tablet, as well as 30 min of observation time with a nurse. For Quebec, no nurse observation time was included in the base case analysis of the ragweed SLIT-tablet because it is not covered by the Régie de l’assurance maladie du Québec. In both Ontario and Quebec, one physician (90% specialist, 10% general practitioner) follow-up visit at the end of the treatment season was assumed for the ragweed SLIT-tablet. For Ontario, each ragweed SCIT injection and MRPATA injection was assumed to be associated with a physician injection administration fee, a physician consultation, and 30 min of observation time with a nurse. For Quebec, no nurse observation time was included in the base case analysis of ragweed SCIT or MRPATA because it is not covered by the Régie de l’assurance maladie du Québec. For the base case analysis for both Ontario and Quebec, it was assumed that 90% of patients visited a specialist and 10% visited a general practitioner for the ragweed SCIT titration visits, 5% visited a specialist and 95% visited a general practitioner for ragweed annual SCIT maintenance visits, and that 20% of patients visited a specialist and 80% visited a general practitioner for the MRPATA visits.

### Model cost inputs

Costs associated with medication and services of healthcare professionals were considered for each treatment included in the analysis. All costs are in Canadian dollars. The costs for the ragweed SLIT-tablet, ragweed SCIT, and MRPATA over a 3-year treatment period for Ontario and Quebec are summarized in Table 2.

**Table 2:** Model cost inputs for medication and services of healthcare professionals in Ontario and Quebec

Cost category	Cost type		$CAD/Unit	
	Ontario	Quebec	Ontario	Quebec
Ragweed SLIT-tablets	Box of 30 tablets		$117.31	$117.31
	Mark-up(27)		8%	6.5%
	Dispensing fee/claim(27, 28)		$8.83	$9.94
Ragweed SCIT vials	Complete Treatment Set—Monovalent standardized [Omega (99100075)] per treatment(25)		$265.00	$265.00
	10 mL maintenance—Monovalent standardized [Omega (02247754)] per vial(25)		$265.00	$265.00
	Complete Treatment Set—Preseasonal Ragweed [Omega (99101150)] per treatment(25)		$265.00	$265.00
MRPATA	MRPATA [Paladin (00464988)] single vial(26)		$111.60	$111.60
	MRPATA [Paladin (00464988)] per treatment (4 vials)		$446.72	$446.72
Physician	Medical specific re-assessment (follow-up visit)—A624(29)	Main visit—(09150 50% institution and 09127 50% private clinic)(30)	$62.05	$93.10
	Partial assessment (pre- or post-injection) with specialist—A628(29)	Control visit—(09152 50% institution and 09129 50% private clinic)(30)	$38.55	$53.38
	Injection (sole reason for visit)—G212(29)	Allergen immunotherapy including professional participation in the process, if necessary, and interpretation—one injection—20105 (50% institution and 50% private clinic)(30)	$9.75	$26.28
	Injection (with consultation at same visit)—G202(29)		$4.45	-
Nurse	Hourly wage (2020)(31)		$56.74	-

*CAD* Canadian dollars, *MRPATA* modified ragweed pollen allergen tyrosine adsorbate, *SCIT* subcutaneous immunotherapy, *SLIT* sublingual immunotherapy

The medication costs were obtained from the manufacturer submitted price for the ragweed SLIT-tablet, from the Régie de l’assurance maladie du Québec [24] for ragweed SCIT, and the Association québécoise des pharmaciens propriétaires [25] for MRPATA. There are multiple inputs for ragweed SCIT costs as there are varied formulations for pre-seasonal and annual treatments. The mark-up for Ontario (8%) and Quebec (6.5%) was obtained from the Patented Medicine Prices Review Board [26]. The dispensing fee for Ontario ($8.83/claim) was obtained from the Ontario Ministry of Health and Long-Term Care [27] and for Quebec ($9.94/claim) was obtained from Patented Medicine Prices Review Board [26]. It was assumed that the costs did not change over the 3-year time horizon of the analysis.

Costs for physician services in Ontario were obtained from the Ontario Schedule of Benefits and Fees [28] and for Quebec were obtained from the RAMQ Manuel des Médecins Spécialistes [29]. Costs for nurse services in Ontario were obtained from the Ontario Nurses’ Association collective agreement (assumes 8 years experience, $48.17/hour plus 4.8% for vacation and 13% for fringe benefits, totaling $56.74/hour) [30].

### Scenario analyses

Several scenario analyses were conducted to determine the impact of many of the key base case assumptions on the outcomes. The different scenarios examined variable discount rates, more MRPATA injections, a lower proportion of specialist titration visits for ragweed SCIT, shorter ragweed SLIT-tablet treatment course, more or less nurse times per SCIT injection (for Ontario only since nursing costs are not covered in Quebec), exclusion of markup and dispensing fees, and addition of nursing costs for Quebec. Scenarios were also considered that assessed the economic impact of the ragweed SLIT-tablet from a societal or patient perspective rather than the payer perspective used in the base case analysis. The patient resources assumed for the societal or patient perspective scenario include patient time lost for office visits and travel distance, details of which are described in Additional file 1: Table S1. The assumed patient costs associated with time lost were an average hourly wage (as of December 2022 for individuals aged 16 years and older) of $30.49 in Ontario and $24.39 in Quebec obtained from Statistics Canada [31]. The assumed cost associated with travel distance by private car for the first 5000 km driven was $0.59 in Ontario and $0.47 per kilometer in Quebec obtained from the Canadian 2020 Reasonable Kilometer-Allowance rates [32].

A scenario that included nurse working time was conducted for Quebec, where it was excluded from the base case scenario because of lack of public plan coverage. For this scenario, the nurse work time in hours was 0 (years 1, 2, and 3 each) for ragweed SLIT-tablet, 5.50 (years 1, 2, and 3 each) for preseasonal SCIT, 15.88 (year 1), 6.50 (year 2), and 6.50 (year 3) for annual SCIT, and 2.0 (years 1, 2, and 3 each) for MRPATA.

## Results

### Costs of ragweed SLIT-tablet and AIT comparators

The total healthcare costs for 3 years of treatment for ragweed SLIT-tablet was $2700.86 in Ontario and $2826.12 in Quebec, for preseasonal ragweed SCIT was $3601.00 in Ontario and $3849.25 in Quebec, for annual ragweed SCIT was $9314.08 in Ontario and $11,576.96 in Quebec, and for MRPATA was $2780.48 in Ontario and $3255.61 in Quebec (Table 3). Yearly costs were the same or similar within each AIT option. Total healthcare costs for all the evaluated AIT options were higher in Quebec than Ontario. Drug costs were the primary cost drivers for the ragweed SLIT-tablet and MRPATA, whereas healthcare professional services were the primary cost drivers for preseasonal and annual ragweed SCIT.

**Table 3:** Yearly and total healthcare costs of ragweed SLIT-tablet, preseasonal ragweed SCIT, annual ragweed SCIT, and MRPATA in Ontario and Quebec ($CAD)

Cost category	Ontario				Quebec			
	Year 1	Year 2	Year 3	Total Years 1–3 (discounted)	Year 1	Year 2	Year 3	Total Years 1–3 (discounted)
Ragweed SLIT-tablet								
Drug costs	$813.12	$813.12	$813.12	$2403.50	$809.23	$809.23	$809.23	$2391.98
Tablet costs	$703.84	$703.84	$703.84	$2080.46	$703.84	$703.84	$703.84	$2080.46
Markup and dispensing fees	$109.29	$109.29	$109.29	$323.04	$105.39	$105.39	$105.39	$311.52
Physician costs	$100.60	$100.60	$100.60	$297.36	$199.85	$119.79	$119.79	$434.14
GP	$59.70	$59.70	$59.70	$176.47	$14.65	$10.64	$10.64	$35.47
Specialist	$40.90	$40.90	$40.90	$120.90	$185.20	$109.14	$109.14	$398.67
**Total healthcare costs**	**$913.72**	**$913.72**	**$913.72**	**$2700.86**	**$1009.08**	**$929.01**	**$929.01**	**$2826.12**
Preseasonal ragweed SCIT								
Drug costs	$295.03	$295.03	$295.03	$872.07	$292.17	$292.17	$292.17	$863.61
Vial costs	$265.00	$265.00	$265.00	$783.31	$265.00	$265.00	$265.00	$783.31
Markup and dispensing fees	$30.03	$30.03	$30.03	$88.77	$27.17	$27.17	$27.17	$80.30
Physician costs	$611.13	$611.13	$611.13	$1806.41	$1010.07	$1010.07	$1010.07	$2985.65
Injection	$117.98	$117.98	$117.98	$348.72	$317.93	$317.93	$317.93	$939.76
GP	$107.25	$107.25	$107.25	$317.02	$289.03	$289.03	$289.03	$854.32
Specialist	$10.73	$10.73	$10.73	$31.70	$28.90	$28.90	$28.90	$85.43
Consultation costs	$493.15	$493.15	$493.15	$1457.69	$692.14	$692.14	$692.14	$2045.89
GP	$80.67	$80.67	$80.67	$238.44	$121.03	$121.03	$121.03	$357.75
Specialist	$412.49	$412.49	$412.49	$1219.26	$571.11	$571.11	$571.11	$1688.14
Nurse costs	$312.09	$312.09	$312.09	$922.51	$0.00	$0.00	$0.00	$0.00
** Total healthcare costs**	**$1218.25**	**$1218.25**	**$1218.25**	**$3601.00**	**$1302.24**	**$1302.24**	**$1302.24**	**$3849.25**
Annual ragweed SCIT								
Drug cost	$590.06	$590.06	$590.06	$1,744.15	$584.33	$584.33	$584.33	$1727.21
Vial costs	$530.00	$530.00	$530.00	$1,566.62	$530.00	$530.00	$530.00	$1566.62
Markup and dispensing fees	$60.06	$60.06	$60.06	$177.53	$54.33	$54.33	$54.33	$160.59
Physician costs	$2284.02	$1873.16	$1873.16	$5947.71	$3793.45	$3096.35	$3096.35	$9849.55
Injection	$396.46	$247.16	$247.16	$879.88	$1068.41	$666.07	$666.07	$2371.16
GP	$309.56	$126.75	$126.75	$557.47	$834.23	$341.58	$341.58	$1502.31
Specialist	$86.90	$120.41	$120.41	$322.41	$234.18	$324.50	$324.50	$868.85
Consultation costs	$1887.56	$1626.00	$1626.00	$5067.83	$2725.04	$2430.28	$2430.28	$7478.38
GP	$963.33	$1545.05	$1545.05	$3985.25	$1445.38	$2318.19	$2318.19	$5979.49
Specialist	$924.24	$80.96	$80.96	$1082.57	$1279.67	$112.09	$112.09	$1498.90
Nurse costs	$900.82	$368.84	$368.84	$1622.22	$0.00	$0.00	$0.00	$0.00
**Total healthcare costs**	**$3774.90**	**$2832.06**	**$2832.06**	**$9314.08**	**$4377.78**	**$3680.68**	**$3680.68**	**$11,576.76**
MRPATA								
Drug cost	$491.29	$491.29	$491.29	$1452.19	$485.70	$485.70	$485.70	$1435.66
Vial costs	$446.72	$446.72	$446.72	$1320.45	$446.72	$446.72	$446.72	$1320.45
Markup and dispensing fees	$44.57	$44.57	$44.57	$131.74	$38.98	$38.98	$38.98	$115.21
Physician costs	$380.45	$380.45	$380.45	$1124.57	$654.68	$654.68	$654.68	$1935.16
Injection	$70.20	$70.20	$70.20	$207.50	$189.18	$189.18	$189.18	$559.19
GP	$39.00	$39.00	$39.00	$115.28	$105.10	$105.10	$105.10	$310.66
Specialist	$31.20	$31.20	$31.20	$92.22	$84.08	$84.08	$84.08	$248.53
Consultation costs	$310.25	$310.25	$310.25	$917.06	$465.50	$465.50	$465.50	$1375.96
GP	$62.05	$62.05	$62.05	$183.41	$93.10	$93.10	$93.10	$275.19
Specialist	$248.20	$248.20	$248.20	$733.65	$372.40	$372.40	$372.40	$1100.77
Nurse costs	$113.49	$113.49	$113.49	$335.46	$0.00	$0.00	$0.00	$0.00
**Total healthcare costs**	**$940.66**	**$940.66**	**$940.66**	**$2780.48**	**$1101.40**	**$1101.40**	**$1101.40**	**$3255.61**

*CAD* Canadian dollars, *GP* general practitioner, *MRPATA* modified ragweed pollen allergen tyrosine adsorbate, *SCIT* subcutaneous immunotherapy, *SLIT* sublingual immunotherapy

Over the total 3-year time horizon, the ragweed SLIT-tablet had a potential cost savings of $900.14 in Ontario and $1023.14 in Quebec when compared with preseasonal ragweed SCIT, of $6613.22 in Ontario and $8750.64 in Quebec when compared with annual ragweed SCIT, and $79.62 in Ontario and $429.49 in Quebec when compared with MRPATA (Table 4). The ragweed SLIT-tablet had higher drug costs compared with the other AIT options, but lower costs for healthcare professional services. The lower costs for healthcare professional services with the ragweed SLIT-tablet were driven by the need for fewer office visits than SCIT.

**Table 4:** Potential healthcare cost savings with the ragweed SLIT-tablet vs preseasonal ragweed SCIT, annual ragweed SCIT, or MRPATA after 3 years of treatment ($CAD)

Cost category	Ontario			Quebec		
	Ragweed SLIT-tablet vs Preseasonal ragweed SCIT	Ragweed SLIT-tablet vs Annual ragweed SCIT	Ragweed SLIT-tablet vs MRPATA	Ragweed SLIT-tablet vs Preseasonal ragweed SCIT	Ragweed SLIT-tablet vs Annual ragweed SCIT	Ragweed SLIT-tablet vs MRPATA
Drug costs	$1531.42	$659.35	$1083.04	$1528.37	$664.76	$1071.52
Physician costs	− $1509.05	− $5,650.35	− $827.20	− $2551.51	− $9415.41	− $1501.02
Nurse costs	− $922.51	− $1,622.22	− $335.46	$0.00	$0.00	$0.00
**Total healthcare costs**	− **$900.14**	− **$6613.22**	− **$79.62**	− **$1023.14**	− **$8750.64**	− **$429.49**

*MRPATA* modified ragweed pollen allergen tyrosine adsorbate, *SCIT* subcutaneous immunotherapy, *SLIT* sublingual immunotherapy

### Scenario analyses

**Fig. 1:**
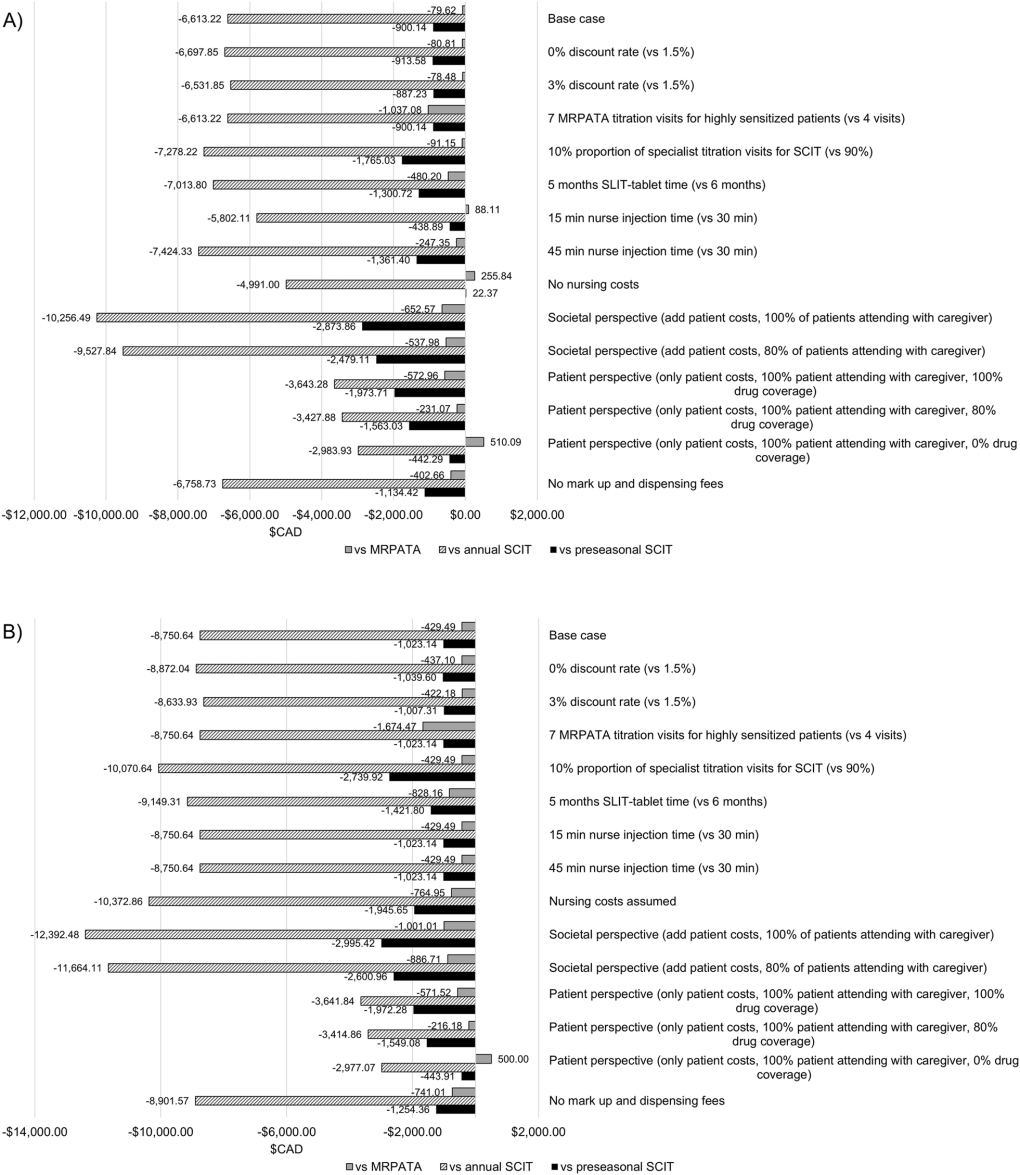
Scenario analyses of potential cost savings after 3 years of treatment with ragweed SLIT-tablet vs preseasonal ragweed SCIT, annual ragweed SCIT, or MRPATA in **A**) Ontario and **B**) Quebec. *MRPATA* modified ragweed pollen allergen tyrosine adsorbate, *SCIT* subcutaneous immunotherapy, *SLIT* sublingual immunotherapy

Results from the scenario analysis indicated that costs were most impacted by including societal costs, which added patient costs and assumed 100% of patients attended visits with a caregiver (Fig. 1A, B). Over the total 3-year time horizon, the ragweed SLIT-tablet from a societal perspective versus the base case had a substantial increase in the potential cost savings. Potential cost savings with the ragweed SLIT-tablet were $2873.86 in Ontario and $2995.42 in Quebec when compared with preseasonal ragweed SCIT, $10,256.49 in Ontario and $12,392.48 in Quebec when compared with annual ragweed SCIT, and $652.57 in Ontario and $1001.01 in Quebec when compared with MRPATA (Fig. 1A, B). There were 3 scenarios for Ontario in which the ragweed SLIT-tablet became more costly than an AIT comparator (vs MRPATA when assuming less nurse time per SCIT injection; vs preseasonal SCIT and MRPATA when assuming no nursing costs; and vs MRPATA when assessing only patient costs and assuming 100% patients attending with a caregiver and 0% drug coverage; Fig. 1A). For Quebec, the only scenario in which the ragweed SLIT-tablet became more costly than an AIT comparator was versus MRPATA when assessing only patient costs and assuming 100% patients attending with a caregiver and 0% drug coverage (Fig. 1B).

## Discussion

The results from this cost minimization analysis indicate that the ragweed SLIT-tablet provides potential cost savings compared with SCIT and MRPATA from a public payer perspective through 3 years of treatment. The cost savings is primarily because fewer clinic appointments are needed with the ragweed SLIT-tablet since it can be self-administered at home. Accordingly, the greatest potential cost savings with the ragweed SLIT-tablet were observed when compared with annual SCIT, which has the greatest number of required clinic visits. MRPATA requires only 4 injection clinic visits per year, and therefore has the smallest difference in cost savings versus the ragweed SLIT-tablet. Greater potential cost-savings with the ragweed SLIT-tablet were observed in Quebec than Ontario, primarily driven by differences in physician costs. Scenario analyses found that the potential cost savings with ragweed SLIT-tablet versus SCIT became even greater from the societal perspective that incorporated the indirect costs of caregiver travel to clinic visits.

Cost minimization analyses have also been conducted with the house dust mite (HDM) SLIT-tablet, 12 SQ-HDM and white birch SLIT-tablet, 12 SQ-Bet in Canada [33, 34]. The analysis for the HDM SLIT-tablet was conducted from a societal perspective in Ontario and Quebec over a 3 year time period, and the comparator was HDM SCIT [33]. Potential cost savings of the HDM SLIT-tablet over HDM SCIT were $1833 in Ontario and $769 in Quebec. Similarly, the analysis for the white birch SLIT-tablet was also conducted from a societal perspective in Ontario and Quebec over a 3 year time period, and the comparator was preseasonal tree SCIT [34]. Potential cost savings of the white birch SLIT-tablet over preseasonal tree SCIT were $1112 in Ontario and $1200 in Quebec. The perspective was the major difference between the current analysis for the ragweed SLIT-tablet and the analyses conducted for the HDM and white birch SLIT-tablets. The HDM and white birch SLIT-tablet base case analyses were from a societal perspective that included patient travel costs and the lost hourly wages associated with clinic visits. Since larger numbers of clinic visits are needed for SCIT than the SLIT-tablets, these indirect patient costs are a key contributor to the cost savings with the SLIT-tablets. Similar findings were observed in the societal perspective scenario in the current ragweed SLIT-tablet analysis, although in this case the indirect cost was for the caregiver since it was assumed that every pediatric patient would be accompanied by an adult caregiver. These indirect costs are not inconsequential for the patient and their families. For example, annual SCIT in the current analysis was assumed to result in 59 h of time spent in just the first year for clinic visits, which at the assumed hourly wage comes to approximately $1800 in lost wages in Ontario and $1400 in Quebec. For patients and their caregivers living in rural areas, the travel time and costs may be an even greater burden. In addition, although there are no associated costs, children have to miss school or after-school activities once a week for the first 6 months during the SCIT titration period and then monthly for subsequent maintenance visits. While it was not possible to quantify the actual patient cost for pediatric patients specifically, some adolescents may have to miss part-time work. Additional opportunity costs that could not be quantified for the patient should also be considered and may be substantial. The at-home administration of the SLIT-tablets has the added advantage of allowing patients to continue their treatment if in-person clinic visits need to be disrupted, as was the case during the early part of the COVID-19 pandemic.

Assumptions for the AIT options had to be made for the analysis and were based on published literature and input from experienced clinical experts. When some of these assumptions were explored in scenario analyses, including the number of MRPATA injections, nurse injection time, or the proportion of SCIT titration visits conducted by specialists versus general practitioners, the impact on the results was minimal. One input that did have a marked impact on the results was nursing costs. In some Canadian provinces, nurse-associated costs in public clinics are a cost for the healthcare system, whereas in private clinics the costs are funded out of the clinic’s earnings. Therefore, in Ontario where nursing costs are covered by the public plan, removing the nursing costs decreased the potential cost savings of the ragweed SLIT-tablet from the public payer perspective. When nursing costs were added into the model for Quebec, where nursing costs are not covered by the public payer plan, there was an increase in the potential cost savings with the ragweed SLIT-tablet.

Ragweed SLIT-tablet treatment in the model had higher drug costs than SCIT or MRPATA. It was assumed in the analysis that the drug costs for the children were covered 100% under their caregiver’s insurance or by a public plan. The scenario analyses indicated that ragweed SLIT-tablet treatment was more costly than MRPATA from the patient perspective when drug coverage was assumed to be 0%, but in this scenario, ragweed SLIT-tablet remained a cost saving alternative to SCIT.

There are some limitations for this cost minimization analysis. First, the model assumptions for the AIT resource use were validated by the clinical experts practicing in Ontario and Quebec, but AIT practice may vary by geographic region. In addition, adherence to treatment was considered to be 100% for all clinic visits and treatments. Adherence to both SLIT and SCIT has been demonstrated to be a challenge, [35, 36] and decreased adherence to clinic visits or the daily use of the SLIT-tablets would impact costs. However, children tend to be more adherent and are less likely to discontinue treatment than adults, possibly because their caregivers have a vested interest in ensuring an optimal outcome [37]. Another limitation is that because of the lack of head-to-head trials, efficacy was assumed to be equal among the AIT options. One study comparing MRPATA and SCIT using aqueous extracts found no improvement of symptoms with MRPATA [38]. Poorer efficacy could result in greater office visits for acute symptoms and greater symptom-relieving pharmacotherapy use, which in turn would increase costs. Another limitation is that the analysis did not consider patients who need AIT for polysensitization to allergens other than ragweed. Use of more than one concurrent SLIT-tablet is not currently approved. Studies have demonstrated the safety of dual SLIT-tablet administration, [39, 40] but efficacy of this practice has yet to be evaluated, and multiallergen SCIT may be needed for such patients.

## Conclusions

In this cost minimization analysis, the ragweed SLIT-tablet provided estimated cost savings from a public payer perspective for the treatment of ragweed ARC in Canadian children compared with SCIT or MRPATA. The potential cost savings with the ragweed SLIT-tablet were observed for both Ontario and Quebec and were maintained in most of the scenario analyses.

## Supplementary Information

**Additional file 1**: **Table S1.** Model patient resource inputs in Ontario and Quebec.

## Data Availability

All data supporting the conclusions of this article are included within the article.

## Abbreviations

AIT: Allergy immunotherapy
ARC: Allergic rhinoconjunctivitis
CAD: Canadian dollars
HDM: House dust mite
MRPATA: Modified ragweed pollen allergen tyrosine adsorbate
SCIT: Subcutaneous immunotherapy
SLIT: Sublingual immunotherapy

## References

[1] Keith PK, Desrosiers M, Laister T, Schellenberg RR, Waserman S (2012). The burden of allergic rhinitis (AR) in Canada: perspectives of physicians and patients. Allergy Asthma Clin Immunol.

[2] Meltzer EO, Blaiss MS, Derebery MJ, Mahr TA, Gordon BR, Sheth KK (2009). Burden of allergic rhinitis: results from the pediatric allergies in America survey. J Allergy Clin Immunol.

[3] Walker S, Khan-Wasti S, Fletcher M, Cullinan P, Harris J, Sheikh A (2007). Seasonal allergic rhinitis is associated with a detrimental effect on examination performance in United Kingdom teenagers: case-control study. J Allergy Clin Immunol.

[4] Bensnes SS (2016). You sneeze, you lose: The impact of pollen exposure on cognitive performance during high-stakes high school exams. J Health Econ.

[5] Sierra-Heredia C, North M, Brook J, Daly C, Ellis AK, Henderson D (2018). Aeroallergens in Canada: distribution, public health impacts, and opportunities for prevention. Int J Environ Res Public Health.

[6] Chan-Yeung M, Anthonisen NR, Becklake MR, Bowie D, Sonia Buist A, Dimich-Ward H (2010). Geographical variations in the prevalence of atopic sensitization in six study sites across Canada. Allergy.

[7] Bousquet J, Schunemann HJ, Togias A, Bachert C, Erhola M, Hellings PW (2020). Next-generation allergic rhinitis and its impact on asthma (ARIA) guidelines for allergic rhinitis based on grading of recommendations assessment, development and evaluation (GRADE) and real-world evidence. J Allergy Clin Immunol.

[8] Roberts G, Pfaar O, Akdis CA, Ansotegui IJ, Durham SR, Gerth van Wijk R (2018). EAACI guidelines on allergen immunotherapy: allergic rhinoconjunctivitis. Allergy.

[9] Ciprandi G, Tosca MA (2021). Under-prescription of allergen-immunotherapy: why is it important to prescribe it in childhood instead?. Immunotherapy.

[10] Cox LS, Murphey A, Hankin C (2020). The cost-effectiveness of allergen immunotherapy compared with pharmacotherapy for treatment of allergic rhinitis and asthma. Immunol Allergy Clin North Am.

[11] ^Pr^Pollinex®-R. (Modified ragweed pollen allergen tyrosine adsorbate pre-filled syringes, vials, suspension for injection). Full Prescribing Information. Allergy Therapeutics (UK) Limited. Worthing, UK. 2017.

[12] Ragwitek. (Standardized allergen extract, short ragweed (*Ambrosia artemisiifolia*) sublingual tablet, 12 Amb a 1-U Canadian monograph). Full Prescribing Information. ALK-Abelló A/S. Hørsholm, Denmark 2020.

[13] Dhami S, Nurmatov U, Arasi S, Khan T, Asaria M, Zaman H (2017). Allergen immunotherapy for allergic rhinoconjunctivitis: A systematic review and meta-analysis. Allergy.

[14] Burks AW, Calderon MA, Casale T, Cox L, Demoly P, Jutel M (2013). Update on allergy immunotherapy: American Academy of Allergy, Asthma & Immunology/European Academy of Allergy and Clinical Immunology/PRACTALL consensus report. J Allergy Clin Immunol.

[15] Canonica GW, Cox L, Pawankar R, Baena-Cagnani CE, Blaiss M, Bonini S (2014). Sublingual immunotherapy: World Allergy Organization position paper 2013 update. World Allergy Organ J.

[16] Chester JG, Bremberg MG, Reisacher WR (2016). Patient preferences for route of allergy immunotherapy: a comparison of four delivery methods. Int Forum Allergy Rhinol.

[17] Damm K, Volk J, Horn A, Allam JP, Troensegaard-Petersen N, Serup-Hansen N (2016). Patient preferences in allergy immunotherapy (AIT) in Germany - a discrete-choice-experiment. Health Econ Rev.

[18] Ellis AK, Boursiquot J, Carr S, Graham F, Masse MS (2020). Patient and physician perceptions of seasonal allergic rhinitis and allergen immunotherapy: a parallel physician patient survey. Allergy Asthma Clin Immunol.

[19] Tankersley M, Winders T, Aagren M, Brandi H, Hasse Pedersen M, Ledgaard Loftager AS (2021). Preference for immunotherapy with tablets by people with allergic rhinitis. Patient Prefer Adherence.

[20] Cox L, Nelson H, Lockey R, Calabria C, Chacko T, Finegold I (2011). Allergen immunotherapy: a practice parameter third update. J Allergy Clin Immunol.

[21] Canadian Agency for Drugs and Technologies in Health (CADTH). Guidelines for the economic evaluation of health technologies.: Canada 2017.

[22] Nolte H, Bernstein DI, Nelson HS, Ellis AK, Kleine-Tebbe J, Lu S (2020). Efficacy and safety of ragweed SLIT-tablet in children with allergic rhinoconjunctivitis in a randomized, placebo-controlled trial. J Allergy Clin Immunol Pract.

[23] Blume SW, Yeomans K, Allen-Ramey F, Smith N, Kim H, Lockey RF (2015). Administration and burden of subcutaneous immunotherapy for allergic rhinitis in U.S. and Canadian clinical practice. J Manag Care Spec Pharm.

[24] Régie de l’assurance maladie du Québec. (Effective September 30, 2020). https://www.ramq.gouv.qc.ca/sites/default/files/documents/liste_med_2022-05-26_en.pdf. Accessed 2 June 2022.

[25] AQPP's medication list.: Association québécoise des pharmaciens propriétaires 2020. https://www.monpharmacien.ca/en/aqpp/aqpps-medication-list/. Accessed 2 June 2022.

[26] Markup policies in public drug plans. Government of Canada; 2020. Available from: http://www.pmprb-cepmb.gc.ca/view.asp?ccid=1312 Accessed 2 June 2022.

[27] Ontario drug benefit program: dispensing fees.: ontario ministry of health and long-term care; 2022. https://www.health.gov.on.ca/en/public/programs/drugs/programs/odb/opdp_dispensing_fees.aspx Accessed 2 June 2022.

[28] Ontario health insurance plan: schedule of benefits and fees (effective October 1, 2021). Ontario ministry of health and long-term care; 2021. https://health.gov.on.ca/en/pro/programs/ohip/sob/ Accessed 2 June 2022.

[29] RAMQ Manuel des Médecins Spécialistes: Rémunération à l’acte: Régie de l’assurance maladie du Québec; 2017. https://www.ramq.gouv.qc.ca/fr/professionnels/medecins-specialistes/manuels/Pages/remuneration-acte.aspx Accessed 2 June 2022.

[30] Ontario Nurses' Association collective agreement 2022. https://www.ona.org/wp-content/uploads/6-20230331_hospcentralagreement-draft.pdf Accessed 2 June 2022.

[31] Average usual hours and wages by selected characteristics, monthly, unadjusted for seasonality (x 1000). Statistics Canada; 2022. http://www.statcan.gc.ca/tables-tableaux/sum-som/l01/cst01/labr69a-eng.htm Accessed 2 June 2022.

[32] Government of Canada 2020 Reasonable per-kilometre Allowance Rates 2020. https://www.canada.ca/en/revenue-agency/services/tax/businesses/topics/payroll/benefits-allowances/automobile/automobile-motor-vehicle-allowances/reasonable-kilometre-allowance.html Accessed 2 June 2022.

[33] Ellis AK, Gagnon R, Hammerby E, Lau A (2019). Sublingual immunotherapy tablet for the treatment of house dust mite allergic rhinitis in Canada: an alternative to minimize treatment costs?. Allergy Asthma Clin Immunol.

[34] Ellis AK, Gagnon R, Hammerby E, Shen J, Gosain S (2021). Sublingual immunotherapy tablet: a cost-minimizing alternative in the treatment of tree pollen-induced seasonal allergic rhinitis in Canada. Allergy Asthma Clin Immunol.

[35] Allam JP, Andreasen JN, Mette J, Serup-Hansen N, Wustenberg EG (2018). Comparison of allergy immunotherapy medication persistence with a sublingual immunotherapy tablet versus subcutaneous immunotherapy in Germany. J Allergy Clin Immunol.

[36] Hsu NM, Reisacher WR (2012). A comparison of attrition rates in patients undergoing sublingual immunotherapy vs subcutaneous immunotherapy. Int Forum Allergy Rhinol.

[37] Malet A, Azpeitia A, Gutierrez D, Moreno F, Moncin Mdel San Miguel M, Cumplido JA (2016). Comprehensive study of patients’ compliance with sublingual immunotherapy in house dust mite perennial allergic rhinitis. Adv Ther.

[38] Hebert J, Small P (1988). Comparison of alum-precipitated aqueous extracts and modified ragweed tyrosine adsorbate vaccine in the treatment of ragweed hay fever. Ann Allergy.

[39] Maloney J, Berman G, Gagnon R, Bernstein DI, Nelson HS, Kleine-Tebbe J (2016). Sequential treatment initiation with timothy grass and ragweed sublingual immunotherapy tablets followed by simultaneous treatment is well tolerated. J Allergy Clin Immunol Pract.

[40] Gotoh M, Okubo K, Yuta A, Ogawa Y, Nagakura H, Ueyama S (2019). Safety profile and immunological response of dual sublingual immunotherapy with house dust mite tablet and Japanese cedar pollen tablet. Allergol Int.

