# Supplementary material for: Minimization of ragweed allergy immunotherapy costs through use of the sublingual immunotherapy tablet in Canadian children with allergic rhinoconjunctivitis
Source: Allergy Asthma Clin Immunol. 2023 Jan 18;19:7. doi: 10.1186/s13223-023-00758-7 (PMC9847451; doi:10.1186/s13223-023-00758-7)
Supplement: Supplementary file 1 — Additional file 1: Table S1. Model patient resource inputs in Ontario and Quebec. [file 13223_2023_758_MOESM1_ESM.docx]

**Additional file Table 1.** Model patient resource inputs in Ontario and Quebec.

| **Resource** | **Ontario and Quebec** | | | | | |
| --- | --- | --- | --- | --- | --- | --- |
|  | **Ragweed SLIT-tablet** | **Preseasonal ragweed SCIT** | **Annual SCIT** | | | **Pollinex-R** |
|  | **Years 1, 2, and 3 each** | **Years 1, 2, and 3 each** | **Year 1** | **Year 2** | **Year 3** | **Years 1, 2, and 3 each** |
| Patient time lost, h | 3.00* | 21.42^†^ | 59.46^†^ | 25.08^†^ | 25.08^†^ | 8.58^†^ |
| Patient travel distance, km^‡^ | 40 | 220 | 635 | 260 | 260 | 80 |

*Travel time round trip, 40 min; wait time per visit, 15 min; first dose observation time, 30 min; physician consultation time, 20 min.

^†^Travel time round trip, 40 min; pre-injection wait time per visit, 15 min; injection time, 5 min; post-injection wait time, 30 min; physician consultation time, 20 min.

^‡^20 km round trip per visit.
